# The reliability of the Arabic version of osteoporosis knowledge assessment tool (OKAT) and the osteoporosis health belief scale (OHBS)

**DOI:** 10.1186/1756-0500-6-138

**Published:** 2013-04-08

**Authors:** Rima M Sayed-Hassan, Hyam N Bashour

**Affiliations:** 1 Department of Internal Medicine, Faculty of Medicine, Damascus University, P.O. Box 9241, Damascus, Syria; 2 Department of Family & Community Medicine, Faculty of Medicine, Damascus University, Damascus, Syria

## Abstract

**Background:**

Knowledge and awareness about osteoporosis and its related risk factors are important contributors to osteoporosis preventive behavior. There is a need to assess the reliability of international osteoporosis-related knowledge and belief measurement tools in Arabic community. This study aimed to assess the reliability of the Arabic version of Osteoporosis Knowledge Assessment Tool (OKAT) and the Osteoporosis Health Belief Scale (OHBS) among Syrian women.

**Methods:**

The study included two phases. The first phase included a forward and backward translation of the osteoporosis-related tools (OKAT and OHBS) followed by a pilot testing. The second phase was an assessment of the test-retest reliability of the tools among a convenience sample of one hundred working women at Damascus Faculty of Medicine and its teaching hospitals. For this purpose each instrument was administered twice to all women at an interval of two weeks. Data collection took place in the fall of 2011, and was facilitated by a trained interviewer whose task was to administer the tools and collect some background data from the women who consented to participate in the study.

**Results:**

A total of one hundred women were recruited in this study for the reliability test-retest of the Arabic version of the tools. The mean age of studied women was 37.1 (SD = 8.4) years. Most of the women were married and nearly one-half of them had a university education. The internal consistency values for OHBS (Cronbach’s alpha = 0.806) as well as the OKAT (Cronbach’s alpha = 0.824) met the 0.7 Cronbach’s alpha value requirement. Item analysis did not necessitate any omissions in either tool. McNemar’s test identified only three items on the OKAT questionnaire that significantly differed from the test to the retest. The OKAT mean score (SD) for the test was 9.4 (2.6) and that for the re-test was 10.1 (2.9). Paired *t* test did not show significant difference (P = 0.068).

**Conclusion:**

The Arabic version of both the Osteoporosis Knowledge Assessment Tool (OKAT) and the Osteoporosis Health Belief Scale (OHBS) was found to be reliable as well as acceptable. Further research is needed as to complete the validation of those tools and to use them at larger scale whether in knowledge assessment or in assessing interventions.

## Background

Osteoporosis is the most common metabolic bone disease, and one of the main causes of mortality and morbidity in the elderly population [1]. It is characterized by low bone mass and micro- architectural deterioration of bone tissue, leading to bone fragility and increased risk of fractures [1-3]. It is widely recognized that osteoporosis is associated with impairments of quality of life following fracture, and increased mortality. The risk of fracture increases approximately 1.5 to 3 fold for each standard deviation decrease in bone mineral density (BMD) [1-4]. Based on these criteria, it has been estimated that 13–18% of women aged 50 and over have osteoporosis, and for those over the age of 80, the proportion rises to 70% [1,4,5].

Osteoporosis is a growing health care problem in developing countries, especially with increasing life expectancy, and it is considered as an important public health concern among aging populations; as the low trauma fractures are associated with premature mortality [6,7]. It seems that bone mineral density is lower in most of the Middle Eastern countries compared to Western countries [6]. However, in the absence of a fracture registry in most Middle Eastern countries, the data available from this region are limited [7]. According to the Technical Report of the World Health Organization, 2.9% of worldwide osteoporosis-related fractures occur in the Eastern Mediterranean countries [1]. The prevalence and incidence of osteoporosis and osteoporotic fractures are unknown in Syria [8].

Osteoporosis is not curable, but it can be prevented in part by increasing the level of physical activity at all ages, cessation of smoking, reduction of alcohol consumption, adequate dietary calcium and vitamin D intake, and fall prevention [2,3]. Numerous studies have found clear association between healthy behaviors or lifestyle and decreased risk of osteoporosis and related fractures [2,5,9]. There is good evidence suggesting that osteoporosis knowledge is one contributor to osteoporosis preventive behavior, though this is not a clear-cut relationship [9-14].

Nothing is known about Syrian women’s health beliefs and their preventative behaviors regarding osteoporosis. Determining their knowledge, beliefs and behaviors about osteoporosis can be helpful in developing effective interventions and guiding public health programs for osteoporosis prevention. Valid and reliable tools to measure osteoporosis-related knowledge and beliefs for Arabic-speaking women are needed for that purpose.

Various tools are available to assess women’s knowledge about osteoporosis. Werner, in 2005, reviewed and analyzed such tools like The Facts on Osteoporosis Quiz (FOOQ), the Osteoporosis Questionnaire (OPQ), The Osteoporosis Knowledge Test (OKT), The Osteoporosis Knowledge Assessment Tool (OKAT). Regardless of the instrument used, serious lacks of knowledge were demonstrated on osteoporosis and its related risk factors [5].

The Osteoporosis Knowledge Assessment Tool (OKAT)developed by Winzenberg and co-authors in 2003; is a 20-item questionnaire, each item having “true”, “false” and “don”t know“ options. Each item is coded 0 if an incorrect answer or a “don’t know” answer is given and 1 if the correct answer was given, with a total potential score of twenty. OKAT focuses on four basic themes: (1) understanding (symptoms and risk of fracture) of osteoporosis (2) the knowledge of risk factors for osteoporosis (3) knowledge of preventive factors as physical activity and diet relating to osteoporosis and (4) treatment availability [10,14].

Healthy life styles and beliefs in child bearing age women may protect them against osteoporosis in later life [1-3], thus measuring women’s beliefs is important. Based on the Health Belief Model, the Osteoporosis Health Belief Scale (OHBS) was developed by Kim and colleagues to measure health beliefs related to osteoporosis [15]. It is a 42-item self-reported questionnaire that was specifically designed to assess beliefs related to calcium intake and exercise behaviors. The instrument consists of seven subscales that measure the perceived susceptibility to osteoporosis, seriousness of osteoporosis, benefits of exercise, benefits of calcium intake, barriers to exercise, barriers to calcium intake, and health motivation. The OHBS uses a 5-point Likert scale to rate the items from strong disagreement (1) to strong agreement (5) [16]. The possible range of scores for each subscale is 6 to 30 with a possible total score range from 42 to 210. For the majority of the subscales, higher scores indicating highly healthy beliefs. But for the two subscales about barriers, higher scores indicate more negative health beliefs.

The purpose of this study was to assess the reliability of the Arabic translation of the Osteoporosis Knowledge Assessment Tool (OKAT) and the Osteoporosis Health Belief Scale (OHBS) among Syrian women. Identifying the most common osteoporosis health beliefs among child bearing Syrian women and assessing their knowledge when planning and conducting education interventions, may be useful in future research as well as in practice for osteoporosis prevention and management.

## Methods

This study was carried out in two phases. Phase one included the translation of the tools. Instruments selected for the reliability assessment were the OHBS and the OKAT. Having reviewed the relevant literature [1-8], OKAT was thought by the researchers (one of them is a rheumatologist) as being the most relevant to the Arabic lay women as it is simple, short, more relevant to the Syrian culture and is compatible with Middle East and North Africa consensus on osteoporosis [6]. OHBS is the most widely used tool to measure health beliefs. The translation process of both OKAT and OHBS from English into Arabic was based on the process of forward and backward translation by two bilingual translators, whose native language is Arabic, and comparison (between the original English version and the back translation version) was completed. A panel of two professional medical translators was involved in the back translation review. An agreement to make the Arabic version more comprehensive was made, thus a footnote including all terms used in Arabic language for Osteoporosis was inserted. The Arabic version of the tools was piloted on twenty women to assure comprehension and ease of administration. Few minor changes were made after the pilot testing. The final Arabic version of both tools (*available from the first author upon request*) went for the second phase of this study. The second phase included an assessment of the test-retest reliability of both tools among a convenience sample of one hundred working women in Damascus, Syria.

Our sample was recruited from the Faculty of Medicine at Damascus University and two of its teaching hospitals as well as from surrounding schools around the campus. The Work law in Syria (Law no 50) allows women with all educational levels to be recruited for different jobs. The main inclusion criteria were apparently healthy women, whose native language is Arabic, and who approved to participate in the study.

For the purpose of the study, each instrument was administered twice to all women at an interval of two weeks after the first implementation. Data collection was facilitated by a trained interviewer whose task was to administer the tools. In addition to the translated OKAT and OHBS, our questionnaire included background characteristics including demographic data, marital status, the number of births, menopausal status, employment, history of drug administration (steroids, thyroid hormone,hormonal therapy,others), history of low-trauma fractures, and socio-economic level. Data collection took place over a period of one month (Sep-Oct, 2011).

Ethical approval for this study was provided by the Institutional Review Board of Damascus University Faculty of Medicine and written informed consent was obtained from each participant.

Data were entered into Microsoft Excel and all statistical analyses were performed using SPSS (Version 19). Descriptive analysis was carried out. The reliability was studied by Cronbach’s alpha to measure the internal consistency and the interclass correlation coefficient for the test-retest reliability. A value of 0.7 was considered acceptable for both the Cronbach’s alpha and the interclass correlation coefficient. If omitting an item significantly increased Cronbach’s alpha, then excluding the item was considered to increase the homogeneity of the scale [17]. The reliability analysis was also performed for sub-categories of OHBS tool. McNemar’s test was used to compare the paired assessment of the OKAT between the test and re-test; thus to identify non-consistent items.

## Results

All women who were approached agreed to participate in the study, and thus we had a response rate of 100%. Table 1 demonstrates the background characteristics of the sampled women. Most of the women were married and nearly one-half of them had a university education. Table 2 presents actual responses for the translated OHBS at test and retest among the study sample. No missing values to any item were reported for all the 42 items. This shows that the contents of the questionnaire were generally acceptable to the respondents. Inter-item correlation as shown in Table 2 was lowest (0.001) for question 21 and highest (0.460) for question 24. As shown in Table 3, the interclass correlation for the test-retest statistic for the OHBS (0.806) was significant. The internal consistency values for the full questionnaire met the 0.7 Cronbach’s alpha value requirement. Item analysis did not necessitate any omissions.

**Table 1:** Women’s characteristics in the study sample (n = 100)*

**Item**	**No**	**%**
Age		
20–29	20	20
30–39	44	44
40–49	27	27
50–51	9	9
**Age Mean (SD)/range**	37.1 (8.4)/23–63	
**BMI Mean (SD)/range**	26.6 (5.5)/18–51	
**Marital Status**		
Single	17	17
Married	81	81
Divorced/Widow	2	2
**Regular menses**		
Yes	75	79
No	20	21
**Age at puberty**		
Less than 13	12	12
13–15	78	78
16+	10	10
**Smoking status**		
Yes	21	21
No	79	79
**History of steroid/hormonal therapy use**		
Yes	22	22
No	78	78
**History of low trauma**		
Yes	13	13
No	87	87
**Work**		
Health professionals	54	54
Non-health professionals	46	46
**Place of work**		
Hospital	64	64
Faculty	17	17
School	19	19
**Education level**		
Up to 9 years of schooling	28	28
10 years of schooling or more	72	72

* Total is not always 100 due to missing values on some items.

**Table 2:** Answers in percentages to the OHBS at test and re-test among the study sample

**Item**	**Test**					**Retest**					
	**SD**	**D**	**N**	**A**	**SA**	**SD**	**D**	**N**	**A**	**SA**	**Inter-item correlation**
1. Your chances of getting osteoporosis are high.	7	37	19	33	4	7	50	12	30	1	0.427
2. Because of your body build, you are more likely to develop osteoporosis.	13	47	14	26	–	7	52	11	30	–	0.291
3. It is extremely likely that you will get osteoporosis.	3	40	18	37	2	7	50	12	31	–	0.199
4. There is a good chance that you will get osteoporosis.	4	37	9	47	3	7	64	13	16	–	0.215
5. You are more likely than the average person to get osteoporosis.	6	54	14	24	2	6	62	16	16	–	0.009
6. Your family history makes it more likely that you will get osteoporosis.	11	64	5	19	1	12	60	7	21	–	0.192
7. The thought of having osteoporosis scares you.	7	31	4	49	9	5	43	7	43	2	0.305
8. If you had osteoporosis you would be crippled.	11	48	15	25	1	5	65	13	16	1	0.261
9. Your feelings about yourself would change if you got osteoporosis.	12	39	13	31	5	5	58	11	24	2	0.198
10. It would be very costly if you got osteoporosis.	9	29	25	32	5	4	52	21	22	1	0.228
11. When you think about osteoporosis you get depressed.	8	44	8	38	2	4	56	11	28	1	0.209
12. It would be very serious if you got osteoporosis.	4	49	6	37	4	2	51	12	31	4	0.228
13. Regular exercise prevents problems that would happen from osteoporosis.	3	26	15	50	6	4	16	21	56	3	0.283
14. You feel better when you exercise to prevent osteoporosis.	1	15	8	67	9	3	7	8	78	4	0.184
15. Regular exercise helps to build strong bones.	1	5	7	62	25	1	7	8	76	8	0.215
16. Exercising to prevent osteoporosis also improves the way your body looks.	2	2	2	60	34	1	1	1	71	26	0.013
17. Regular exercise cuts down the chances of broken bones.	3	19	17	47	14	1	30	12	54	3	0.269
18. You feel good about yourself when you exercise to prevent osteoporosis.	–	7	8	71	14	2	6	7	79	6	0.347
19. Taking in enough calcium prevents problems from osteoporosis.	2	9	3	74	12	3	8	3	82	4	0.196
20. You have lots to gain from taking in enough calcium to prevent osteoporosis.	–	10	8	72	10	3	15	3	75	4	0.108
21. Taking in enough calcium prevents painful osteoporosis.	–	17	16	60	7	2	25	12	58	3	0.001
22. You would not worry as much about osteoporosis if you took in enough calcium.	1	16	10	63	10	2	24	4	65	5	0.165
23. Taking in enough calcium cuts down on your chances of broken bones.	–	11	7	74	8	3	28	5	59	5	0.096
24. You feel good about yourself whenyou take in enough calcium to prevent osteoporosis.	–	6	7	73	14	1	11	7	75	6	0.460
25. You feel like you are not strong enough to exercise regularly.	4	26	5	61	4	4	33	5	56	2	0.188
26. You have no place where you can exercise.	2	36	2	52	8	6	50	4	37	3	0.303
27. Your spouse or family discourages you from exercising.	11	32	5	45	7	6	32	5	54	3	0.182
28. Exercising regularly would mean starting a new habit which is hard for you to do.	3	24	6	58	9	5	36	5	51	3	0.362
29. Exercising regularly makes you uncomfortable.	20	54	6	17	3	10	39	9	40	2	0.087
30. Exercising regularly upsets your every day routine.	7	46	6	38	3	8	44	5	41	2	0.193
31. Calcium rich foods cost too much.	14	58	5	22	1	7	73	6	14	–	0.300
32. Calcium rich foods do not agree with you.	13	71	3	12	1	12	72	5	11	–	0.100
33. You do not like calcium rich foods.	13	63	3	19	2	15	62	9	14	–	0.180
34. Eating calcium rich foods means changing your diet which is hard to do.	12	55	7	24	2	13	53	9	25	–	0.252
35. In order to eat more calcium rich foods you have to give up other foods that you like.	12	57	6	20	5	13	60	6	20	1	0.351
36. Calcium rich foods have too much cholesterol.	6	43	15	32	4	5	46	7	32	–	0.298
37. You eat a well-balanced diet.	3	25	9	57	6	1	15	13	71	–	0.308
38. You look for new information related to health.	3	10	7	72	8	1	11	11	75	2	0.118
39. Keeping healthy is very important for you.	2	2	–	60	36	1	4	3	70	22	0.094
40. You try to discover health problems early.	3	35	6	45	11	4	26	9	57	4	0.205
41. You have a regular health check-up even when you are not sick..0.942.	16	47	5	29	3	4	32	8	54	2	0.251
42. You follow recommendations to keep you healthy.	3	29	9	50	9	2	20	4	70	4	0.294

SD = Strongly Disagree, D = Disagree, N = Neutral, A = Agree, SA = strongly agree.

**Table 3:** Internal consistency of the OHBS for all participants and test-retest reliability for all participating subjects

**OHBS**	**No. of items**	**Cronbach’s alpha**	**Inter class correlation (95% CI)**
Susceptibility of osteoporosis (Q 1–6)	6	0.778	0.778
			(0.709–0.838)
Seriousness (Q 7–12)	6	0.822	0.822
			(0.766–0.870)
Benefits of exercises (Q 13–18)	6	0.739	0.739
			(0.656–0.808)
Benefits of calcium intake (Q 19–24)	6	0.749	0.749
			(0.669–0.816)
Barriers to exercises (Q 25–30)	6	0.744	0.744
			(0.663–0.812)
Barriers to calcium intake (Q 31–36)	6	0.716	0.716
			(0.692–0.828)
Health motivation (Q 37–42)	6	0.766	0.766
			(0.627–0.792)
All Items (Q 1–42)	42	0.806	0.806
			(0.748–0.857)

Table 4 presents the responses to the translated OKAT questionnaire at test and retest among the study sample. The percent of correct answers as shown was noted by one of the authors (RSH) who is a rheumatologist and whom reviewed the medical consensus regarding all items. It was evident that items 2, 14 and 18 differed between the test and re-test administration of the questionnaire. McNemar’s test showed a statistical significance differences for those items. Reliability statistics for all the 20 items of the translated OKAT was high (Cronbach’s alpha = 0.824), and the interclass correlation for the test-retest statistic (0.824) was significant (p < 0.001).

**Table 4:** Correct answers for the OKAT at test and re-test among the study sample

**Item**	**% Correct answers at test**	**% Correct answers at re-test**	
1. Osteoporosis leads to an increased risk of bone fractures.	98%	94%	1.0
2. Osteoporosis usually causes symptoms (e.g. pain) before fractures occur.	9%	27%	0.002
3. Having a higher peak bone mass at the end of childhood gives no protection against the development of osteoporosis in later life.	27%	29%	0.557
4. Osteoporosis is more common in men.	88%	85%	0.344
5. Cigarette smoking can contribute to osteoporosis.	49%	44%	0.167
6. White women are at highest risk of fracture as compared to other races.	48%	50%	0.227
7. A fall is just as important as low bone strength in causing fractures.	47%	43%	1.0
8. By age 80, the majority of women have osteoporosis.	82%	82%	0.508
9. From age 50, most women can expect at least one fracture before they die.	55%	66%	0.267
10. Any type of physical activity is beneficial for osteoporosis.	30%	45%	0.061
11. It is easy to tell whether I am at risk of osteoporosis by my clinical risk factors.	48%	47%	0.690
12. Family history of osteoporosis strongly predisposes a person to osteoporosis.	58%	51%	0.442
13. An adequate calcium intake can be achieved from two glasses of milk a day.	68%	75%	0.845
14. Sardines and broccoli are good sources of calcium for people who cannot take dairy products.	53%	51%	0.041
15. Calcium supplements alone can prevent bone loss.	58%	56%	0.327
16. Alcohol in moderation has little effect on osteoporosis.	18%	23%	0.383
17. A high salt intake is a risk factor for osteoporosis.	41%	39%	0.332
18. There is a small amount of bone loss in the ten years following the onset of menopause.	4%	31%	0.000
19. Hormone therapy prevents further bone loss at any age after menopause.	42%	30%	0.200
20. There are no effective treatments for osteoporosis available in “Syria”*.	25%	39%	0.210

*Originally Australia [10].

Further a score for OKAT was calculated using the code of 0 for an incorrect answer or a “don’t know” answer and the code 1 if the correct answer was given. The mean score (SD) for the test was 9.4 (2.6) and that for the re-test was 10.1 (2.9). Paired *t* test did not show significant difference (P = 0.068). As expected, the mean score of OKAT was significantly higher among health professional women as compared to the non-professionals (P = 0.017). No other characteristic of the women was correlated with the score.

## Discussion

This study aimed to test the reliability of the Arabic version of the tools commonly used to assess the knowledge and beliefs related to osteoporosis. We found that the selected tools (OKAT and OHBS) for our study were reliable and had an overall strong interclass correlation reflecting stability over time (two weeks) and across most of the items.

In general, the OHBS in our study met the statistical requirement for the reliability issue. The degree of consistency for OHBS was comparable to other studies [18-20] in spite of cultural differences of Arab women.

OKAT covers the core knowledge about osteoporosis, and is very useful as a baseline as well as follow up measurement when implementing educational interventions. The Arabic version of OKAT showed consistent findings between the test and retest except for three questions (namely; 2, 14, 18). We could explain this by possible improvement in our sample’s knowledge after the first measurement, or lack of clarity and vagueness in some questions, or even slight difficulty in the questions. Question eighteen was only answered by 6% of Australian women where the tool was originally implemented [10]. We do not think that translation errors are responsible for this lack of consistency, as we used a professional panel of medical translators to review the back translation. However one also might argue that specific items in the tool might be less relevant in our culture and religious context where women, for example, do not practice regular exercise or do not appreciate different advantages of physical activity. Furthermore, the question related to exercises (question 10) was better answered by Australian women [10]. No attempt was made to ask participants whether they sought information between the test and re-test due to ethical considerations as this was not part of the informed consent. In spite of all above, the degree of consistency for OKAT was comparable to other studies [21]. In general, the findings indicate poor knowledge of Osteoporosis among the sample. The mean score during the test was less than 10, referring to knowledge of less than 50% of the questions.

A major limitation of this study is that its sample is a convenience one and rather small. A sample of 171 would have been ideal for a minimum acceptable reliability of 0.6 according to rules for reliability studies [22]. Further, the sample does not represent the general population of women in Syria, where women are less educated as compared to ours [23]. However, we have no reason to argue that the implementation of the tool to another group with different characteristics would impact the reliability of the tool, except for the fact that a self-administered application of the tools would be impossible among illiterate women, and an interview may have to be used, making logistics more difficult. In the light of our current findings, we feel that implementing those reliable tools using a larger and more representative sample is important. Discriminant analysis, which was not carried out in this work, will be of value then.

Having OHBS and OKAT translated to Arabic, and proven reliable is of a great value. The burden of osteoporosis may be greater in developing countries, including the Middle East [6-8] where most of Arab women live. One can argue that women in the Arab world may be more prone to osteoporosis for obvious cultural and social reasons such as greater number of pregnancies, longer lactation, low physical activity and potentially lower vitamin D due to both inadequate exposures to sunlight as well as dress style, and possibly reduced vitamin D and calcium intake [6,24]. With this note, we have only selected women in our sample, assuming that reliability check for men should not be different.

In an effort to move forward with implementation of the tools among Arab speaking women and actual assessment of the Osteoporosis-related knowledge and beliefs, the authors will use the tools in a community-based setting and on a large sample of Syrian women.

## Conclusion

The Arabic version of the OHBS and OKAT shown to be reliable tools for assessing women’s knowledge and behavior regarding osteoporosis and its risk factors in the Syrian setting. This study was only a reliability study, and the translated versions of the OKAT and OHBS into Arabic was easily understood by participants and were reliable tools. Further research is needed as to complete the validation of those tools and to use them at larger scale whether in knowledge assessment or in assessing interventions.

## Competing interests

The authors declared that they have no competing interests.

## Authors’ contributions

RSH conceived the study, participated in the design, supervised data collection and drafted the manuscript. HB participated in the design, performed the statistical analysis and drafted the manuscript. Both authors read and approved the final manuscript.
